# Leveraging Predictive Pharmacometrics-Based Algorithms to Enhance Perinatal Care—Application to Neonatal Jaundice

**DOI:** 10.3389/fphar.2022.842548

**Published:** 2022-08-11

**Authors:** Gilbert Koch, Melanie Wilbaux, Severin Kasser, Kai Schumacher, Britta Steffens, Sven Wellmann, Marc Pfister

**Affiliations:** ^1^ Pediatric Pharmacology and Pharmacometrics, University Children’s Hospital Basel (UKBB), University of Basel, Basel, Switzerland; ^2^ NeoPrediX AG, Basel, Switzerland; ^3^ Division of Neonatology, University Children’s Hospital Basel (UKBB), University of Basel, Basel, Switzerland; ^4^ Department of Neonatology, Hospital St. Hedwig of the Order of St. John of God, University Children’s Hospital Regensburg (KUNO), University of Regensburg, Regensburg, Germany

**Keywords:** algorithm, prediction, jaundice, hyperbilirubinemia, mechanism-based modeling

## Abstract

The field of medicine is undergoing a fundamental change, transforming towards a modern data-driven patient-oriented approach. This paradigm shift also affects perinatal medicine as predictive algorithms and artificial intelligence are applied to enhance and individualize maternal, neonatal and perinatal care. Here, we introduce a pharmacometrics-based mathematical-statistical computer program (PMX-based algorithm) focusing on hyperbilirubinemia, a medical condition affecting half of all newborns. Independent datasets from two different centers consisting of total serum bilirubin measurements were utilized for model development (342 neonates, 1,478 bilirubin measurements) and validation (1,101 neonates, 3,081 bilirubin measurements), respectively. The mathematical-statistical structure of the PMX-based algorithm is a differential equation in the context of non-linear mixed effects modeling, together with Empirical Bayesian Estimation to predict bilirubin kinetics for a new patient. Several clinically relevant prediction scenarios were validated, i.e., prediction up to 24 h based on one bilirubin measurement, and prediction up to 48 h based on two bilirubin measurements. The PMX-based algorithm can be applied in two different clinical scenarios. First, bilirubin kinetics can be predicted up to 24 h based on one single bilirubin measurement with a median relative (absolute) prediction difference of 8.5% (median absolute prediction difference 17.4 μmol/l), and sensitivity and specificity of 95.7 and 96.3%, respectively. Second, bilirubin kinetics can be predicted up to 48 h based on two bilirubin measurements with a median relative (absolute) prediction difference of 9.2% (median absolute prediction difference 21.5 μmol/l), and sensitivity and specificity of 93.0 and 92.1%, respectively. In contrast to currently available nomogram-based static bilirubin stratification, the PMX-based algorithm presented here is a dynamic approach predicting individual bilirubin kinetics up to 48 h, an intelligent, predictive algorithm that can be incorporated in a clinical decision support tool. Such clinical decision support tools have the potential to benefit perinatal medicine facilitating personalized care of mothers and their born and unborn infants.

## Introduction

The field of medicine is undergoing a fundamental change in which artificial intelligence is connecting with diagnostic instruments, patient information systems and therapy management enabling unforeseen opportunities in transforming the health system towards a modern data-driven patient-oriented approach (Rajkomar et al., 2019). This paradigm shift also affects perinatal medicine as predictive algorithms and artificial intelligence are applied to enhance and individualize maternal, neonatal and perinatal care, with the goal not only to predict mortality (Mangold et al., 2021) but also to facilitate therapeutic decisions for our most vulnerable patients, fetuses and newborns, and their mothers.

In this work, we discuss a predictive algorithm in neonatology, with initial focus on hyperbilirubinemia, a medical condition affecting half of all newborns. Hyperbilirubinemia is a condition defined as elevated serum or plasma bilirubin levels above the reference range of the laboratory, and it is due to disorders or immaturity of bilirubin metabolism. In neonates, transient jaundice is a normal part of postnatal transition (Dennery et al., 2001). Bilirubin has strong antioxidant properties but when reaching too high levels, bilirubin can cross the blood-brain barrier and might cause bilirubin-induced neurotoxicity, of which kernicterus is the most dangerous form (Watchko and Tiribelli, 2013). Thus, medical screening of all neonates for hyperbilirubinemia is recommended to commence prompt therapy, namely phototherapy, once certain thresholds are crossed to prevent neurological complications (Watchko and Tiribelli, 2013). Up to 10% of neonates experience rebound hyperbilirubinemia, requiring re-initiation of treatment (So and Khurshid, 2021), and making hyperbilirubinemia the major reason for re-hospitalization in the first year of life (Schiltz et al., 2014).

Currently, static population-based nomograms for the assessment of neonatal hyperbilirubinemia are applied in daily clinical practice (Dennery et al., 2001). These nomograms are based on percentiles of bilirubin values at a given age in hours and classify neonates into risk groups. More recent risk stratification approaches include additional clinical factors for the prediction of neonatal hyperbilirubinemia shortly after birth (Castillo et al., 2018) or before discharge (Han et al., 2015). Even though approaches for risk stratification provide clinicians with a guideline for their assessment, adherence is only 50% due to cumbersome documentation (Tartaglia et al., 2013; Sampurna et al., 2018). Moreover, it has been found that health care professional noncompliance with best practices is the main reason for kernicterus in countries with highest health care standards (Alkén et al., 2019). Nomogram-based methods are overly general and do not provide an individual prediction of what will happen. Therefore, we aim for personalized prediction by identifying neonates at risk for clinically relevant hyperbilirubinemia more accurately, thus preventing the development of severe neonatal jaundice as well as overtreatment and unnecessary hospital stays.

Machine learning (ML) methods are computationally powerful tools for the analysis of large and heterogeneous datasets almost in real time (Koch et al., 2020a). Such methods can be applied for discriminating between classes or patient populations (e.g. high *vs*. low risk patient; treatment required yes or no) by identifying relevant variables (features) of interest. As such we recently developed a ML-based tool to predict the probability of whether a neonate will need a phototherapy treatment or not within the next 48 h (Daunhawer et al., 2019). Although this ML tool provides an innovative risk assessment regarding phototherapy requirement, this algorithm does not predict the dynamics of bilirubin kinetics, i.e., this ML algorithm is not able to predict bilirubin levels up to 24 h or 48 h.

Complementary to our previously published ML-based algorithm, we present a predictive PMX-based algorithm (Koch et al., 2020b) that computes individual bilirubin kinetics up to 48 h. The PMX-based algorithm is intended for non-intensive care units to facilitate and optimize management of neonates with jaundice supporting clinical decisions such as 1) is an additional bilirubin measurement necessary? 2) can the neonate be discharged home? 3) can a neonate at risk for clinically relevant hyperbilirubinemia be identified early?

This manuscript has five objectives. First, we describe the development of the PMX-based algorithm. Second, we define clinically relevant scenarios, i.e., prediction up to 24 h based on one bilirubin measurement, and prediction up to 48 h based on two or more bilirubin measurements, and validate the prediction of developed PMX-based algorithm, which is the main goal of this manuscript. An appropriate external validation is a crucial step to perform predictions at the individual patient level. Third, we carry out stress test of this algorithm with increased prediction horizons up to 60 h. Fourth, we assess the sensitivity and specificity of the developed algorithm to evaluate performance relevant to clinical practice in neonatology. Further, we discuss opportunities and challenges of applying “intelligent” ML-, artificial neural networks (ANN)- and PMX-based algorithms in the field of perinatal medicine.

## Methods

This section is structured as follows. First, we explain the magnitude of total serum bilirubin (TSB) measurement errors in clinical practice. Second, we present the study patient populations applied for development and validation of the PMX-based algorithm. Third, we describe the development of the PMX-based algorithm to characterize postnatal bilirubin kinetics. Fourth, we present the development of the PMX-based algorithm to predict individual bilirubin kinetics. Fifth, we outline the validation procedure of the developed PMX-based algorithm. Sixth, we provide information on applied software for descriptive statistics, algorithm development and validation.

### Magnitude of Total Serum Bilirubin Measurement Errors in Clinical Practice

TSB measurements are subject to considerable intra- and inter-individual variability due to biological factors and measurement errors related to clinical practice and laboratory measurements. Van Imhoff et al. (van Imhoff et al., 2011) showed that the inter-laboratory variability was up to a CV of 14.1%. Hence, anticipated magnitude of variability associated with TSB measurements in clinical practice is expected to be of the order of 5–15%.

### Study Patients

#### Dataset for PMX-Based Algorithm Development

The dataset for model development (University Children’s Hospital Basel, Basel, Switzerland) comprises TSB measurements from neonates admitted directly after birth to the neonatal unit due to varying reasons such as respiratory morbidity, birth complications, infection, mild prematurity and feeding problems. None of the neonates suffered from inherited diseases such as glucose-6-phosphate dehydrogenase (G6PDH) deficiency. All neonates included in this study had an inconspicuous neurological status, including those with values in the further course exceeding 15 mg/dl. The bilirubin measurements prior to phototherapy available in this dataset and used for model development consisted of 1,478 measurements from 342 patients, see Table 1 for more details. All bilirubin measurements were performed as total bilirubin using an ABL800 FLEX blood gas analyzer (Radiometer Medical ApS, Denmark). The study was approved by the Institutional Review Board (EKNZ:BASEC 2018-00053).

**TABLE 1:** Key characteristics of the dataset for algorithm development (Basel, Switzerland) and validation (Regensburg, Germany). Values are presented as follows: Median [Q1, Q3] (Min, Max).

Gestational Age (week + day)	Weight at Birth (gram)	Delivery Mode (C-section *vs*. Vaginal Delivery)	Postnatal hour of Last Bilirubin Measurement
Basel, Switzerland (342 neonates with 1,478 bilirubin values, average 4.3 values per neonate)			
37 + 6 [34 + 1, 39 + 5] (32 + 0, 42 + 5)	2,500 [1,950, 3,400] (1,050, 5,520)	179 C.S. 163 Vaginal	77 [56,124] (1, 411)
Regensburg, Germany (1,101 neonates, 3,081 bilirubin values, average 2.8 values per neonate)			
38 + 2 [36 + 2, 39 + 6] (24 + 0, 42 + 2)	3,085 [2,532, 3,580] (520, 5,015)	620 C.S. 481 Vaginal	87.2 [63.0, 115.3] (1, 359)

#### Dataset for External PMX-Based Algorithm Validation

The dataset for external algorithm validation (University Children’s Hospital Regensburg, Hospital St. Hedwig of the Order of St. John, Regensburg, Germany) comprises TSB measurements in two clinical settings: 1) 80% healthy neonates staying with their mothers after birth until discharge home (the majority) or until admission to the neonatal unit because of significant neonatal hyperbilirubinemia or other reasons, 2) 20% neonates admitted after birth to the neonatal unit due to varying reasons such as respiratory diseases, birth complications, infection, mild prematurity and feeding problems. The goal was to apply and validate the PMX-based algorithm in these two clinical settings to cover various neonatal medical conditions and a wide range of postnatal bilirubin time courses. Some neonates suffered from blood group incompatibility; details of which were not reported. All neonates included in this study had an inconspicuous neurological status, including those with values in the further course exceeding 15 mg/dl. Of note, healthy neonates staying with their mothers after birth obtained the bilirubin check together with the mandatory metabolic screening at day 2 or 3 of life. Timing of bilirubin measurement was individualized based on medical or practical factors representing clinical workflow in a perinatal center. Bilirubin measurements prior to phototherapy in this dataset were utilized for model validation, see Table 1 for more details. All bilirubin measurements were performed as total bilirubin utilizing a Bilimeter 3D (Pfaff medical GmbH, Germany). The study was approved by the ethics commission of the University of Regensburg (21-2,518-104).

### Development of PMX Model to Characterize Postnatal Bilirubin Kinetics

**FIGURE 1:**
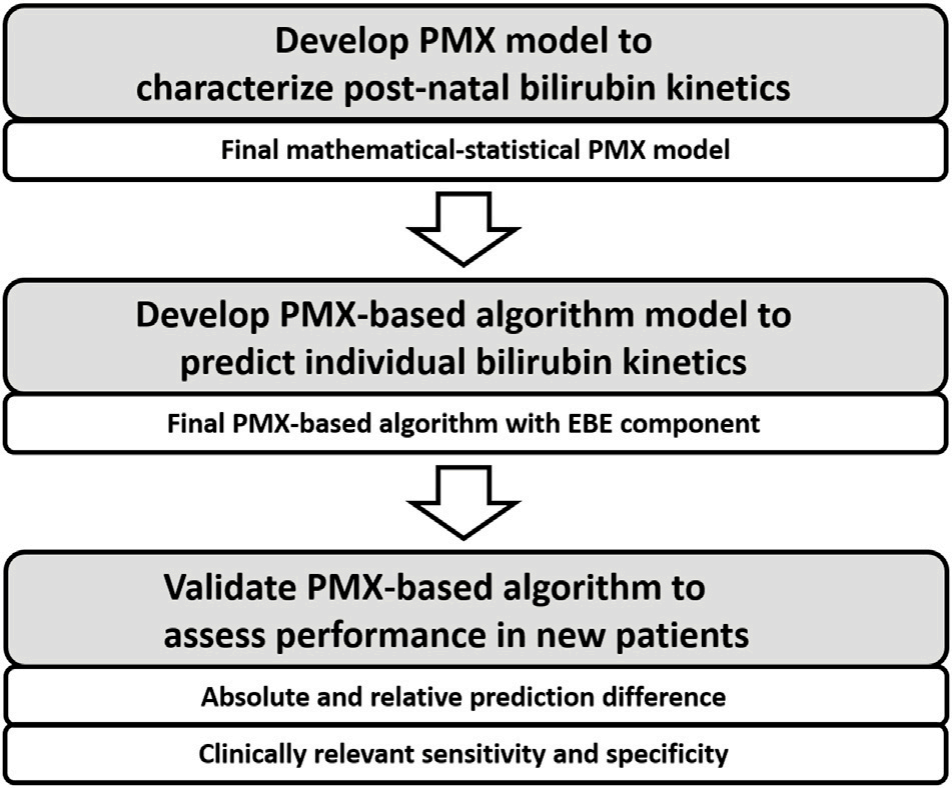
Workflow of the three components for model development and validation.

In this section, the development process of the PMX-based algorithm to characterize individual bilirubin kinetics is presented, compare Figure 1. First, develop structure of the mathematical-statistical PMX model to characterize postnatal bilirubin kinetics based on physiological mechanisms. Second, apply a non-linear mixed effects modeling approach (Lavielle, 2014) to fit the dataset for development to estimate the fixed and random effects resulting in the mathematical-statistical model.

#### Structure of Mathematical Model to Characterize Bilirubin Kinetics

In healthy individuals beyond the neonatal period, most physiological processes are in an equilibrium, i.e., in balance between production and elimination. Consequently, this results in constant bilirubin levels. For neonates shortly after birth, the equilibria of many processes are not yet reached due to maturation. Hence, bilirubin production might be increased, and elimination might be reduced during the first days of life, leading to elevated bilirubin levels. This physiological principle of bilirubin levels B(t) is modeled with a differential equation consisting of a zero-order production term $k_{prod}$ and a first-order elimination term $k_{elim}$ (Dayneka et al., 1993; Koch et al., 2013; Koch and Schropp, 2018)

$$\frac{d}{dt}B\left(t\right)=k_{prod}\left(t,\theta ,c,\beta \right)-k_{elim}\left(t,\theta ,c,\beta \right)\cdot B\left(t\right),\;\;\;B\left(0\right)=B^{0}$$ (1)

where t is postnatal age (PNA), $B^{0}$ is the initial condition (i.e., a parameter for bilirubin level at birth), θ the structural model parameters, c the covariates (i.e., patient characteristics such as birth weight, gestational age and delivery mode) and β the parameters characterizing the covariate effect on the model parameters. The detailed mathematical model structure is part of a broader active patent (Koch et al., 2020b) where more information on Eq. (1) can be found. The structural model parameters are summarized in

$$\text{Θ}=\left(\theta ,B^{0}\right).$$

#### Data Fitting and Development of Mathematical-Statistical Model

The non-linear mixed effects modeling approach was applied for data fitting and parameter estimation. Briefly, structural model parameters have a population value ${\text{Θ}}_{pop}$ (also called fixed effect or typical value) describing the average patient in the population. To characterize an individual neonate in the population, individual model parameters are drawn from a normal distribution with covariance matrix Ω (called random effects). The normal distribution is further transformed to a log-normal distribution to allow log-normally distributed individual model parameters, see (Lavielle, 2014) for more technical details. In addition, the parameter β characterizing covariate effects is estimated. Typically, only covariate effects that show 1) a statistically significant effect, 2) a reduced objective function value, 3) a reduced variability of the random effects, and 4) are clinically relevant and routinely available in clinical practice, are included. Finally, the developed mathematical-statistical model is given by Eq. (1) together with fixed and random effects

$$\rho =\left({\text{Θ}}_{pop},\text{Ω},\beta \right)$$ (2)

### Development of PMX-Based Algorithm to Predict Individual Bilirubin Kinetics

In this section, the development process of the PMX-based algorithm to predict individual bilirubin kinetics is presented, compare Figure 1. The final PMX-based algorithm with an Empirical Bayesian Estimation (EBE) component is applied to predict the individual bilirubin kinetics for a new patient.

#### Final PMX-Based Algorithm to Predict Individual Bilirubin Kinetics

The mathematical-statistical model defined by (Eqs 1, 2) is the final (trained) model based on the dataset applied for development. To predict the bilirubin kinetics for a new patient, EBE, also known as Maximum A Posteriori Estimation (Bassett and Deride, 2019), is applied. The EBE utilizes Eq. (1) and the prior information stored in ρ Eq. (2) about the population applied for model development and training, and estimates the individual model parameters ${\hat{\text{Θ}}}_{i}$ for a new patient by minimizing

$${\hat{\text{Θ}}}_{i}=\text{argmin}\;\left\{-2\log\text{p}\left({\text{Θ}}_{i}\text{|}w_{i};\rho \right)\right\}\;$$ (3)

based on the new individual bilirubin measurements $w_{i}$ and patient characteristics. These estimated individual model parameters ${\hat{\text{Θ}}}_{i}$ are then utilized to perform the individual prediction of bilirubin kinetics.

#### Implementation of PMX-Based Algorithm

Model development was performed in the NLME software The Monolix Suite 2020 (Lixoft, Orsay, France). Since The Monolix Suite 2020 is a commercial software that does not allow application in app- or web-based tools, the developed mathematical-statistical model (Eqs 1, 2) and the EBE Eq. (3) was re-implemented in Matlab 2021 (MathWorks, Natick, MA, USA).

### Validation of PMX-Based Algorithm

In this section, the application and validation of the PMX-based algorithm is presented. First, definitions of the different validation scenarios and some input rules are shown. Second, validation metrics are given, including the absolute und relative prediction difference as well as clinically relevant sensitivity and specificity, compare Figure 1. Third, the construction of validation datasets is briefly discussed.

#### Definition of Validation Scenarios and Input Rules

In the following, clinically relevant validation scenarios are defined for prediction horizons up to 24 and 48 h. To provide a *stress test* for the PMX-based algorithm, additional validation scenarios with longer prediction horizons were also included.

##### Definition of Validation Scenario 1: Prediction up to 24 h Based on One TSB Measurement

PMX-based algorithm predicts for one TSB measurement the bilirubin kinetics for up to 24 h with respect to the time point of the measurement.

##### Definition of Validation Scenario 2a: Prediction up to 48 h Based on Two TSB Measurements

PMX-based algorithm predicts for two TSB measurements the bilirubin kinetics for up to 48 h with respect to the time point of the second measurement.

##### Definition of Validation Scenario 2b: Prediction up to 48 h Based on Two or More TSB Measurements

PMX-based algorithm predicts for two or more TSB measurements the bilirubin kinetics for up to 48 h with respect to the time point of the last measurement.

##### Definition of Stress Test Scenarios With Longer Prediction Horizon

The prediction horizon for one TSB measurement (validation scenario 1) was extended by an additional 6 h, i.e., for a total prediction of up to 30 h. The prediction horizon for two, (validation scenario 2a), or two or more (validation scenario 2b) TSB measurements were extended by an additional 12 h, i.e., for a total prediction of up to 60 h.

##### Definition of Input Rules Regarding Postnatal Age

The time point of the first TSB measurement must be between ≥ 8 and ≤ 72 h of PNA. All further time points of TSB measurements must be between ≥ 24 and ≤ 96 h of PNA. The PNA distance between successive measurements must be ≥ 8 h.

#### Definition of Validation Metrics

##### Definition of Absolute Prediction Difference and Relative Prediction Difference

The absolute prediction difference (p.d.) between predicted bilirubin level $B_{pred}$ and measured (observed) bilirubin level $B_{obs}$ was defined as

$$p.d.=\left|\;B_{pred}-B_{obs}\right|$$ (4)

The relative (absolute) prediction difference (r.p.d.) in percent was defined as

$$r.p.d.=\;\frac{\left|\;B_{pred}-B_{obs}\right|}{B_{obs}}\cdot 100$$ (5)

##### Definition of Clinically Relevant Sensitivity and Specificity

For validation, e.g., of diagnostic tests and algorithms with a binary outcome, statistical measures such as sensitivity and specificity are essential. As such we define these performance measures for our developed PMX-based algorithm in the context of a clinically relevant bilirubin threshold in neonatology. The phototherapy limit for the most vulnerable late preterm and term born neonates is 15 mg/dl (equals to 250 μmol/l) when older than 72 h (Bhutani, 2011). As such this bilirubin level has been set as the threshold to evaluate the performance of the PMX-based predictive algorithm. It should be noted that a bilirubin level > 250 μmol/l is considered clinically relevant, requiring appropriate monitoring and management. Moreover, for a neonate with hyperbilirubinemia, an under-prediction with a value below the threshold would possibly lead to inadequate therapeutic management depending on the magnitude of under-prediction. Taking into account variability in the prediction, e.g., caused by measurement errors, an acceptance range for the prediction difference $B_{pred}-B_{obs}$ is defined by applying the Bland-Altman method (Altman and Bland, 1983) with 5th and 95th percentile of the standard normal distribution (corresponding to 90% limits of agreement):

$$acceptance\;range=\left[MW_{diff}-1.6449\cdot SD_{diff}\;,\;MW_{diff}+1.6449\cdot SD_{diff}\right]$$

where $MW_{diff}$ is the mean of the prediction differences, and $SD_{diff}$ is the standard deviation of the prediction differences.

This defines a criterion for clinically interchangeably measurements and allows for characterizing accepted true positives or accepted true negatives, respectively.

The following terms are defined:

1) True positive: Neonate with hyperbilirubinemia (i.e., observed bilirubin level > 250 μmol/l) either with a predicted bilirubin level > 250 μmol/l or with both a predicted bilirubin level ≤ 250 μmol/l and a prediction difference within the acceptance range (accepted true positive)
2) True negative: Neonate without hyperbilirubinemia (i.e., observed bilirubin level ≤ 250 μmol/l) either with a predicted bilirubin level ≤ 250 μmol/l or with both a predicted bilirubin level > 250 μmol/l and a prediction difference within the acceptance range (accepted true negative)
3) False positive: Neonate without hyperbilirubinemia with a predicted bilirubin level > 250 μmol/l but with a prediction difference above the upper limit of the acceptance range
4) False negative: Neonate with hyperbilirubinemia with a predicted bilirubin level ≤ 250 μmol/l but with a prediction difference below the lower limit of the acceptance range

**FIGURE 2:**
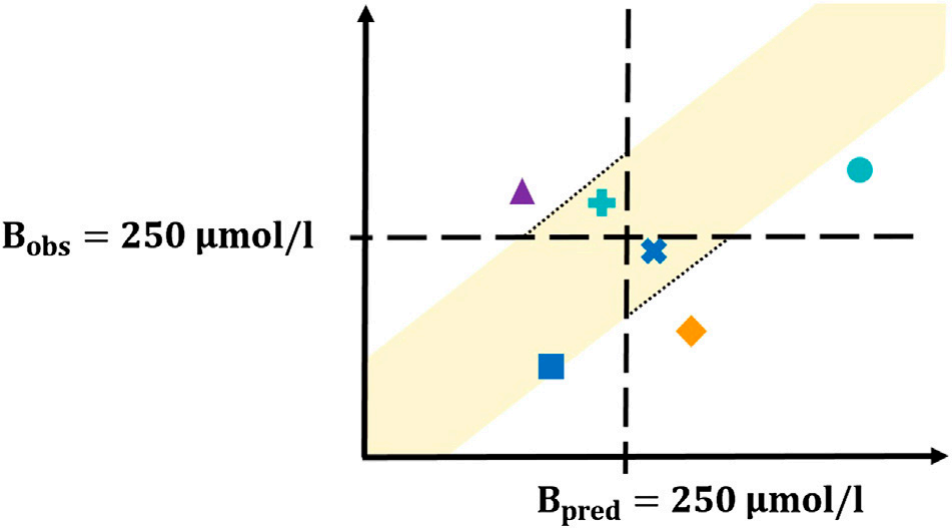
Concept plot for sensitivity/specificity calculation. The four different colors correspond to the four possible test results. The yellow shaded area corresponds to the acceptance range, the turquoise dot and plus represent the true positives, the blue square and cross display the true negatives, the orange diamond corresponds to the false positives and the purple triangle represents the false negatives. Detailed explanation is provided in the main text.

Based on these terms, sensitivity and specificity measures were calculated. The four situations (test results) (i)-(iv) are conceptually visualized in Figure 2 and explained in the following. The black dashed horizontal and vertical lines correspond to a bilirubin level of 250 μmol/l. The yellow shaded area displays the acceptance range. Situation (i), the area of true positives, is shown with turquoise shapes. The dot represents a neonate with hyperbilirubinemia with $B_{pred}>250\;\text{μmol}/\text{l}$ and the plus represents a neonate with hyperbilirubinemia with $B_{pred}\leq 250\;\text{μmol}/\text{l}$ but with a prediction difference in the acceptance range, indicated by the black dotted line. For the true negatives, situation (ii), the analogous situation is given in blue. The square corresponds to a neonate without hyperbilirubinemia with $B_{pred}\leq 250\;\text{μmol}/\text{l}$ and the cross corresponds to a neonate without hyperbilirubinemia with $B_{pred}>250\;\text{μmol}/\text{l}$ but with a prediction difference within the acceptance range, again indicated by the black dotted line. The two remaining situations are the false positives, situation (iii), displayed with orange diamonds, and the false negatives, situation (iv), displayed with purple triangles.

#### Construction of Validation Datasets

The initial number of neonates in the validation dataset was *n* = 1,101. After deletion of patients with exactly one bilirubin measurement and patients with partially missing values, n = 892 neonates were available for the validation data set.

##### Construction of Validation Datasets for One Measurement (Scenario 1) With Prediction Horizon up to 24 h

From the *n* = 892 neonates, eligible neonates for this scenario were selected as follows. The first bilirubin measurement served as user input based on the input rules regarding PNA. Time point of the second measurement was tested to determine whether it fulfills the ≤24 h PNA distance with respect to the first measurement. If yes, all additional measurements were deleted, and this neonate is identified as eligible for the validation dataset, if, in addition all other input rules are met as well. This resulted in a validation dataset which consists of *n* = 236 neonates. Please note that the second measurement is the bilirubin level that will be predicted. In addition, a stress test validation dataset with a prediction horizon up to 30 h instead of 24 h was similarly constructed resulting in *n* = 387 neonates.

##### Construction of Validation Datasets for Two (Scenario 2a) and Two or More (Scenario 2b) Measurements With Prediction Horizon up to 48 h

In these scenarios, only neonates with three or more measurements were eligible. Construction of validation datasets was a step-by-step procedure. First, the PNA distance between the second and the last measurement was computed. Second, if this PNA distance fulfills ≤48 h, then this measurement was selected to be predicted. If the PNA distance is larger, the last measurement was rejected and the PNA distance between the second and the second last measurement was computed, and the procedure was repeated. For the validation datasets with two bilirubin measurements, all measurements between the second and the measurement selected to be predicted were deleted. For the validation datasets with two or more bilirubin measurements, these values were kept. Finally, all input rules were tested and neonates that do not fulfill the input rules were deleted. The final validation sets for scenario 2a (two bilirubin measurements) consist of *n* = 119 neonates and for scenario 2b (two or more bilirubin measurements) consist of *n* = 111 neonates. The stress test validation datasets for two or two or more bilirubin measurements with a prediction horizon up to 60 h was constructed with a similar procedure resulting in *n* = 132 and *n* = 122 neonates, respectively.

### Software Applied for Descriptive Statistics, Algorithm Development and Validation

Descriptive statistical analysis was carried out in R 3.6.0 (R core team, Vienna, Austria). Non-linear mixed effects modeling for model development was performed in The Monolix Suite 2020 (Lixoft, Orsay, France). Construction of validation datasets was performed in R. Model validation was conducted in Matlab 2021 (MathWorks, Natick, MA, USA). A-posteriori data visualization was implemented in R and Matlab.

## Results

This section is structured as follows. First, results of the PMX-based algorithm development is presented. Second, results of the external validation are shown.

### Development of PMX-Based Algorithm to Predict Individual Bilirubin Kinetics

Development of the PMX-based algorithm to predict individual bilirubin kinetics is presented. First, results regarding data fitting and model parameter estimation are briefly given. Second, the verification of the EBE implementation in Matlab is shown.

#### Data Fitting and Model Parameter Estimation (Fixed and Random Effects)

**FIGURE 3:**
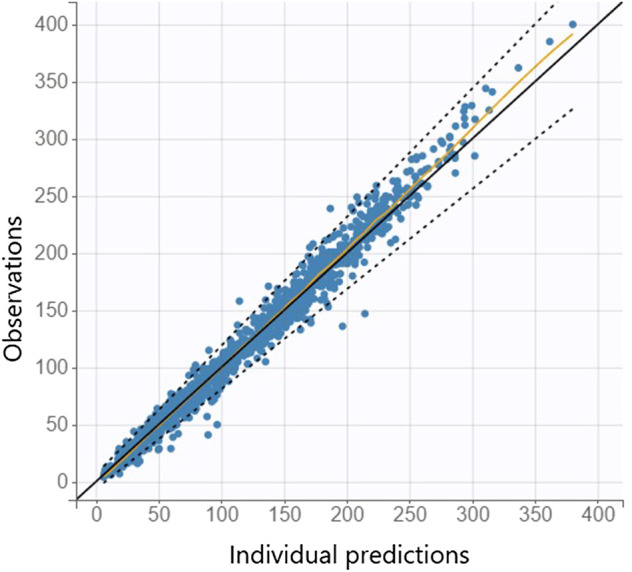
Individual observation vs. prediction plot is shown from the mathematical-statistical model based on the dataset for model development where the orange line indicates the spline and dashed lines the 90% prediction interval.

The mathematical model Eq. (1) was fitted to the dataset for model development resulting in estimates for the fixed and random effects, as well as covariate effects Eq. (2). Several covariates such as gestational age, sex, delivery mode, Apgar scores, arterial pH, weight (at birth and progression), hemoglobin, sodium, hematocrit, feeding (formula, mother milk), Rh blood group system and blood type of mother and neonate, and maternal factors were tested, compare (Daunhawer et al., 2019) and see Supplementary Table S1 in the supplemental material for more details. Weight at birth (continuous), gestational age (continuous) and delivery mode (categorical) were statistically significant covariates and included in the final model with typical PMX covariate approaches. The observation vs. prediction plot of the mathematical-statistical model (Eqs 1, 2) with dataset for model development is shown in Figure 3 **.**

#### Verification of EBE Implementation in Matlab

To verify the EBE implementation in Matlab, each individual neonate from the dataset for model development was re-fitted in Matlab and individual model parameter estimates were compared with the results from *Monolix*. Since the model parameters have different magnitudes, the percent difference over all model parameters was calculated. Comparison of model parameters obtained from Monolix and Matlab showed a median maximal discrepancy of 0.48% caused by non-identical, but structurally similar numerical algorithms applied in both software programs, as well as internal tolerances and termination criteria settings.

### External Validation of the PMX-Based Algorithm

**FIGURE 4:**
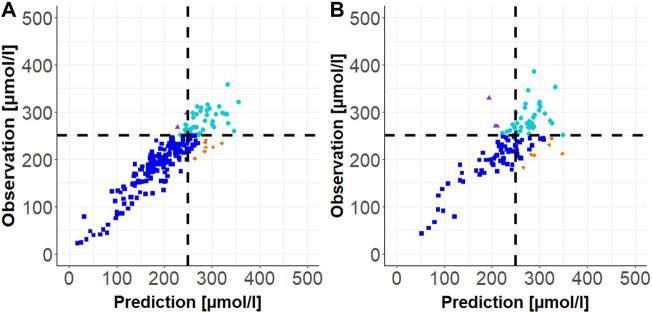
Individual observation vs. prediction plot for Scenario 1 (one TSB measurement) in **(A)** and for Scenario 2a (two TSB measurements) in **(B)**. The dashed black lines correspond to the phototherapy limit of 250 μ mol/l; turquoise dots display true positives, blue squares display true negatives, orange diamonds display false positives and purple triangles display false negatives.

Results of the external validation for scenarios 1, 2a, and 2b as well as the stress test validation are presented as follows. In Table 2, median of the relative (absolute) prediction difference, median of the absolute prediction difference, and the sensitivity and specificity are shown. Observation versus prediction plots for scenario 1 (one measurement with prediction horizon up to 24 h) and scenario 2a (two measurements with prediction horizon up to 48 h) are shown in Figure 4.

**TABLE 2:** For each scenario (including the stress tests), the median of relative (absolute) prediction difference (r.p.d.) Eq. (5), the median of absolute prediction difference (p.d) Eq. (4), and the sensitivity and specificity are presented.

Scenario	Median of r.p.d. in Percent (%)	Median of p.d. mg/dl (µmol/l)	Sensitivity/Specificity	Prediction Horizon
Scenario 1 (one TSB meas.)	8.5%	1.0 mg/dl (17.4 μmol/l)	95.7%/96.3%	Up to 24 h
Scenario 1 (one TSB meas. stress test)	7.9%	0.9 mg/dl (15.7 μmol/l)	92.5%/97.5%	Up to 30 h
Scenario 2a (two TSB meas.)	9.2%	1.3 mg/dl (21.5 μmol/l)	93.0%/92.1%	Up to 48 h
Scenario 2a (two TSB meas. stress test)	9.9%	1.3 mg/dl (22.3 μmol/l)	91.7%/94.0%	Up to 60 h
Scenario 2b (two or more TSB meas.)	8.8%	1.2 mg/dl (20.5 μmol/l)	94.6%/93.2%	Up to 48 h
Scenario 2b (two or more TSB meas. stress test)	9.3%	1.3 mg/dl (21.8 μmol/l)	92.7%/93.8%	Up to 60 h

## Discussion

As perinatal medicine is undergoing a fundamental change transforming towards a modern data-driven patient-oriented approach new tools that will predict the dynamics of biomarkers for an individual fetus or newborn will become increasingly important in maternal, neonatal and perinatal care. We developed PMX-based algorithms to optimize and individualize dosing of therapeutics in the field of perinatal medicine (Wilbaux et al., 2016a; Wilbaux et al., 2016b; Koch et al., 2017; Nekka et al., 2017; van Donge et al., 2018; Dallmann et al., 2019; van Donge et al., 2019; Wilbaux et al., 2019; van Donge et al., 2020a; van Donge et al., 2020b; Koch et al., 2020c; Dao et al., 2020; Samiee-Zafarghandy et al., 2022). It is time to go beyond classical pharmacological applications and develop algorithms condensing the wealth of clinical data and physiology knowledge into predictive tools coping with the dynamics of biomarkers for an individual fetus or neonate.

These tools can be developed based on various methods, such as PMX-based mathematical-statistical computer models, machine learning (ML) or other artificial intelligence (AI) methods such as artificial neural networks (ANNs). In the following, PMX, ML and ANN approaches are discussed with focus on our perinatal case study.

The undisputed major advantage of ML methods is its computational efficiency in handling big data (Koch et al., 2020a). Large amounts of input features can be processed regarding its relationship with a dependent variable, e.g., a labeled (supervised) binary outcome. On one hand this allows large amounts of input features to be screened, e.g., patient characteristics, for their relevance, but on the other hand the ML-based tool is solely data-driven. Recently, we developed a ML-based tool to predict the probability whether a neonate will need a phototherapy treatment or not within the next 48 h (Daunhawer et al., 2019). Almost 50 features were screened resulting in a relevant subset of only four, which suffices for a strong predictive performance (Daunhawer et al., 2019). Although such ML-based tool provides an innovative risk assessment regarding phototherapy requirement, it does not predict the dynamics of bilirubin kinetics. In addition, ML methods are not pre-destined to represent physiological mechanisms. Hence, we consider ML as a powerful tool e.g., in pre-screening large amounts of input features and in developing diagnostic tools where dynamic aspects of the dependent variable are not of primary importance.

AI methods, such as ANNs, have become popular to analyze data from various fields as ANNs can approximate any function up to a certain accuracy (Hornik et al., 1989). At first glance, this sounds like the perfect tool to learn any kind of behavior. Although this is true in theory, an ANN is solely data-driven, i.e., anything the ANN will learn arises from the analysis dataset which can have essential fundamental consequences. An enormous amount of data may be required covering all possible situations. What ANNs do not see, will not be learned, and may not be accurately predicted. Another issue with ANNs is its black-box property, which makes it almost impossible to understand why a trained ANN looks the way it does. This in turn can limit acceptance of ANN-based algorithms by care givers in clinical practice.

The developed PMX-based algorithm presented in this paper includes known physiology-based, biological, and clinical facts (Bonate, 2006; Koch et al., 2013; Gabrielsson, 2017). As an example, neonates undergo strong maturation processes during the first days and even weeks of life. We think it is “intelligent” to incorporate such scientific, medical understanding into our computer models. Our PMX-based algorithm predicts bilirubin kinetics over time up to 48 h. Hence, not only an answer for a specific question is available for the clinician (Koch et al., 2020a), but the entire bilirubin kinetics is revealed and provided. In addition, due to the availability of the predicted bilirubin kinetics, different clinical end points of interest can be defined in an a-posteriori step, e.g., prediction up to 24 h, 48 h or even longer prediction horizons, as presented in this paper. Moreover, clinically relevant binary end points such as prediction above or below a certain threshold, can be defined, as presented in the sensitivity and specificity computations.

Discussed PMX-, ML- and ANN-based methods have in common that an external validation, i.e., a dataset from another medical center, is necessary before application in clinical practice. The major goal of this paper was to present an external validation of the PMX-based algorithm based on a dataset that was not available during algorithm development. In addition to typical validation procedures in pharmacometrics (Lavielle, 2014), we applied the statistical concept of sensitivity and specificity for the external validation of the PMX-based algorithm. This is to demonstrate that one can translate PMX-based algorithms that forecast dynamics of biomarker responses or disease progression into simplified algorithms that predict a binary outcome.

The developed, predictive PMX-based algorithm was applied in two different clinically relevant scenarios in neonatology. In the first scenario, bilirubin kinetics is predicted up to 24 h into the future based on a single bilirubin measurement with a median relative (absolute) prediction difference of 8.5% (median absolute prediction difference 17.4 μmol/l), and sensitivity and specificity of 95.7 and 96.3%, respectively. In the second scenario, bilirubin kinetics is predicted up to 48 h into the future based on two bilirubin measurements with a median relative (absolute) prediction difference of 9.2% (median absolute prediction difference 21.5 μmol/l), and sensitivity and specificity of 93.0 and 92.1%, respectively. Moreover, a scenario with two or more bilirubin measurements and various stress tests based on increasing the prediction horizon were also performed. In all these cases, similar values regarding the applied validation metrics were obtained.

Recently, the PMX-based algorithm has even been validated with three additional external, independent datasets: 1) clinical dataset from Greece consisting of neonates with transcutaneous bilirubin (TcB) measurements only, 2) clinical dataset from Germany consisting of neonates with TSB only, TcB only, or combinations of TSB and TcB measurements, and 3) clinical dataset from Kenya, Africa, consisting of neonates with TSB and TcB measurements. Results from these additional external validation studies will be published in the near future.

Until now, best practice has been to plot measured bilirubin values to given nomograms in a paper or electronic-based fashion to identify the current level of patients’ jaundice status. Then, for estimating individual risk of a given neonate and to provide a recommendation for next measures, including further bilirubin controls or specific therapy management steps, various clinical parameters need to been considered by the responsible health care provider. As long as the neonatal patient is hospitalized anyway there is only the medical challenge. In contrast, once there is no other reason for keeping the patient in hospital or the patient is already in the outpatient service, clinical decision making becomes even more demanding as additional organizational, economic and legal challenges may arise (Brown et al., 2021). However, there are no randomized and quasi-randomized studies available specifically addressing bilirubin therapy, namely home-versus hospital-based phototherapy (Malwade and Jardine, 2014).

Our intelligent PMX-based algorithm for prediction of bilirubin kinetics is based on differential equations that characterize maturation processes and other balance properties and are then trained and validated on large datasets. PMX-based algorithms can complement “artificial intelligence” such as ML- and ANN-based approaches in perinatal medicine. PMX-based algorithms leverage and integrate scientific, medical knowledge with intelligent learning from clinical data. Our developed intelligent algorithm for bilirubin level prediction will be incorporated in a clinical decision support tool with the goal to further optimize and individualize treatment of preterm and term neonates, our most vulnerable patients. The presented case of hyperbilirubinemia illustrates the potential of intelligent, predictive ML-, ANN- or PMX-based algorithms in neonatology.

There are numerous opportunities for such clinical decision support tools to further enhance and personalize care of mothers and their unborn and born children. Neonatal jaundice is just one of many medical conditions affecting newborn babies. There are many other diseases in fetuses, neonates and their mothers rooted in the specific dynamics of pregnancy and transition from intra-uterine to extra-uterine life (Evers and Wellmann, 2016). In contrast to adult medicine where health is defined as a continuum and the absence of physical and mental degradation, in perinatal medicine, health is a matter of cycles, growth, development and maturation processes. As such intelligent algorithms and tools designed for predicting medical conditions in perinatal medicine must address these specific properties.

## Data Availability

The raw data supporting the conclusions of this article will be made available by the authors, without undue reservation.
