# Supplementary material for: Leveraging Predictive Pharmacometrics-Based Algorithms to Enhance Perinatal Care—Application to Neonatal Jaundice
Source: Front Pharmacol. 2022 Aug 11;13:842548. doi: 10.3389/fphar.2022.842548 (PMC9402995; doi:10.3389/fphar.2022.842548)
Supplement: Supplementary file 1 [file DataSheet1.pdf]

Version: June 6, 2022

Supplemental material

for

Leveraging predictive pharmacometrics-based algorithms to enhance perinatal care – application to neonatal jaundice

Table S1: Covariates of the 342 patients from the dataset applied for development of PMX-based algorithm. In case of missing covariate, number of patients with available covariate is explicitly indicated.

	Unit	Median [Q1,Q3]	Min, Max	Proportion
Gestational age	wk+day	37+6 [34+1, 39+5]	32+0, 42+5	
Sex	female			44% (n = 149)
Delivery mode	vaginal			48% (n = 163)
Apgar 1	---	7 [5, 8]	0, 10	
Apgar 5	---	8 [7,10]	0, 10	
Arterial pH (n = 289)	---	7.27 [7.20, 7.32]	6.88, 7.50	
Birth weight	g	2500 [1950, 3400]	1050, 5520	
Weight max loss	%	-4.5 [-6.3, -2.7]	-18, 0	
Extensive weight loss > 10%	yes			3.8% (n = 13)
Hemoglobin*	g/l	186 [170, 203]	122, 249	
Sodium* (n = 331)	mmol/l	136 [134, 138]	125, 148	
Hematocrit* (n = 245)	%	57 [52, 62]	37, 75	
Rh blood group system (n = 240)	positive			89% (n=213)
Blood type (n = 197)				0 33% (n = 65)<br>A 45% (n = 89)<br>AB 9% (n = 17)<br>B 13% (n = 26)
Feeding formula	yes			34% (n = 116)
Feeding mother milk	yes			84% (n = 286)
Mother age	y	32 [29, 35]	17, 47	
Mother rh blood group system	positive			85% (n = 290)
Mother blood type (n = 315)				0 32% (n = 100)<br>A 46% (n = 145)<br>AB 8% (n = 24)<br>B 14% (n = 46)

* determined at first bilirubin measurement
